# Casticin Inhibits A375.S2 Human Melanoma Cell Migration/Invasion through Downregulating NF-κB and Matrix Metalloproteinase-2 and -1

**DOI:** 10.3390/molecules21030384

**Published:** 2016-03-19

**Authors:** Zih-Yun Wu, Jin-Cherng Lien, Yi-Ping Huang, Ching-Lung Liao, Jen-Jyh Lin, Ming-Jen Fan, Yang-Ching Ko, Yu-Ping Hsiao, Hsu-Feng Lu, Jing-Gung Chung

**Affiliations:** 1 Department of Biological Science and Technology, China Medical University, No. 91, Hsueh-Shih Road, Taichung 40402, Taiwan; mary28862001@gmail.com; 2 School of Pharmacy, China Medical University, Taichung 40402, Taiwan; jclien@mail.cmu.edu.tw; 3 Department of Physiology, China Medical University, Taichung 40402, Taiwan; yphuang@mail.cmu.edu.tw; 4 School of Chinese Medicine, China Medical University, Taichung 40402, Taiwan; qbking@ms29.hinet.net; 5 Division of Cardiology, China Medical University Hospital, Taichung 40402, Taiwan; pig222@ms15.hinet.net; 6 Department of Biotechnology, Asia University, Taichung 40402, Taiwan; mjfan@asia.edu.tw; 7 Institute of Clinical Medical Science, China Medical University, Taichung 40402, Taiwan; kyc621006@yahoo.com.tw; 8 Department of Dermatology, Chung Shan Medical University Hospital, Taichung 40402, Taiwan; cs1601@csmu.edu.tw; 9 Department of Restaurant, Hotel and Institutional Management, Fu-Jen Catholic University, New Taipei 112, Taiwan; 10 Departments of Clinical Pathology, Cheng Hsin General Hospital, No. 45, Cheng Hsin St., Taipei 112, Taiwan

**Keywords:** casticin, MMP-2, migration, invasion, A375.S2 cells

## Abstract

Casticin is one of the main components from *Fructus Viticis*, which is widely used as an anti-inflammatory agent. The mechanism of how casticin affects melanoma cell migration and invasion is still not well known. Here we studied the anti-metastasis effects of casticin on A375.S2 melanoma cells by using a non-lethal concentration. First; we used an adhesion assay to test the A375.S2 cells’ adhesion ability after treatment with casticin. We next investigated the cell migration ability after casticin treatment by using a wound healing assay to prove that the migration of A375.S2 cells can be inhibited by casticin and double checked the results using the transwell-migration assay. The suppressive effects on matrix metalloproteinase-2; and -9 (MMP-2; and -9) activities were examined by gelatin zymography. Furthermore, western blotting was used to investigate the protein level changes in A375.S2 cells. We found that p-EGFR; Ras and p-ERK1/2 are decreased by casticin, indicating that casticin can down-regulate the migration and invasion ability of A375.S2 cells via the p-EGFR/Ras/p-ERK pathway. The NF-κB p65 and p-ERK levels in nuclear proteins are also decreased by treatment with casticin. An EMSA assay also discovered that the NF-κB p65 and DNA interaction is decreased. NF-κB p65 protein level was examined by immunofluorescence staining and also decreased. Our findings suggest that casticin has anti-metastatic potential by decreasing the invasiveness of A375.S2 cells. We also found that casticin suppressed A375.S2 cell proliferation and cell adhesion ability, but did not affect cell death, as examined using cytometry and a collagen adhesion assay. Based on these observations, casticin could be used as an inhibitor of migration and invasion of human melanoma cells in the future.

## 1. Introduction

Cancer metastasis accounts for more than 90% of all cancer-related deaths and it has been reported to be a multistep process including cell adhesion, migration and invasion [1,2,3]. Inhibiting cancer cell metastasis is one of the strategies for the cancer therapy and research [4]. Tumor invasion and migration is an indispensable process for cancer metastasis, and requires proteolytic degradation of the extracellular matrix (ECM) and basement membrane of normal surrounding tissues by a family of zinc-dependent proteolytic enzymes named matrix metalloproteinases (MMPs) [5,6]. It was reported that MMP expression is increased in a variety of cancer types, where it is indicative of invasive disease and leads to difficult clinical prognosis [7]. MMPs were reported to involve cell motility and adhesion [8] and metastasis [9]. Among MMPs, MMP-2 and MMP-9 in particular, have been recognized to play critical roles in the degradation of ECM proteins in the basement membrane [10,11]. It was also reported that high levels of MMP-2 and MMP-9 expression are associated with inflammation, tissue repair, cancer progression, invasion, and metastasis [12]. Therefore, the suppression of MMPs, including MMP-2/9, and decreasing cancer cell invasion and migration are one of the strategies for preventing cancer cell metastasis.

Traditional Chinese Medicine has used the fruit of *Vitex rotundifolia* L. which grows in the countrysides of China, Taiwan, and Korea [13], for treating gastroenteritis, inflammation, and headaches [14,15]. Many investigators are interested in investigating the components of medicinal herbs used in complementary and alternative medicines [16]. Casticin, vitexilactone, viteagnusin I, and several vitetrifolins (D-G) have been isolated from *Vitex rotundifolia* [17] and it was reported that casticin (a polymethoxyflavone) exerts multiple biological and pharmacological activities [18,19]. Casticin is one of the active ingredients derived from *Fructus Viticis* [20] and it inhibited acute inflammation in a mouse model [21], induced cervical cancer cell apoptosis through reactive oxygen species-mediated mitochondrial signaling pathways [22], induced cytocidal effects against the human promyelocytic cell line HL-60 [23], induced gastric cancer cell apoptosis through endoplasmic reticulum stress [24] and suppressed self-renewal and invasion of lung cancer stem-like cells from A549 cells through down-regulation of pAKT [25]. Casticin was also one of the ingredients from Vitex agnuscastus which have been shown to exhibit a potent lipoxygenase inhibition [26] and also inhibited monocyte oxidative burst [27]. Casticin was isolated from *Vitex negundo* and shown to inhibit cell cycle progression at G2/M phase and induce apoptosis in mammalian cancer cells [28].

Recently, it was reported that casticin inhibits COX-2 and iNOS expression via suppression of NF-κB and MAPK signaling in lipopolysaccharide-stimulated mouse macrophages [29]. Casticin may thus have therapeutic potential in inflammatory lung diseases, such as chronic obstructive pulmonary disease (COPD) [30]. Casticin suppressed migration of eosinophil and expression of chemokines and adhesion molecules in A549 lung epithelial cells via NF-κB inactivation [31]. Although casticin has been reported to exert anti-oxidant, anti-inflammatory, and anticancer activities, there is no available information to show casticin inhibits cancer cell migration and invasion in human melanoma A375.S2 cells *in vitro*. Herein, we show the first evidence of the anti-migration and-invasion activity of casticin on A375.S2 cells. This study demonstrates that casticin is a potent anti-metastasis agent against human melanoma cells through the signaling mechanisms of the NF-κB and MAPK pathways in A375.S2 cells.

## 2. Results

### 2.1. Casticin Decreased the Cell Viability of A375.S2 Cells

**Figure 1:**
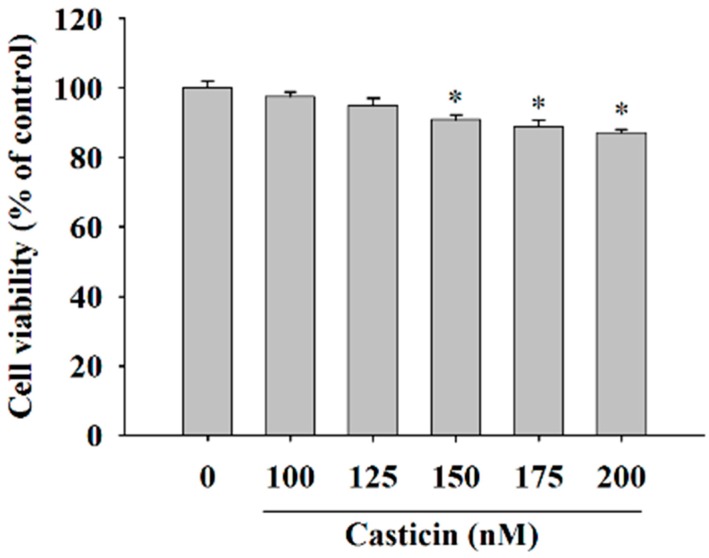
Casticin affects the percentage of viable A375.S2 cells. Cells (5 × 10^4^ cells/well) were incubated with 0, 100, 125, 150, 175 and 200 nM for 24 h before cells were harvested and the percentage of viable cells were determined by flow cytometry as described in the Materials and Methods Section. * *p* < 0.05, significant difference between casticin-treated groups and the control as analyzed by Student’s *t* test.

In order to investigate the cytotoxicity of casticin on A375.S2 cells, we treated them with casticin (0, 100, 125, 150, 175 and 200 nM) for 24 h before the cells were collected for viability assay and the results are shown in Figure 1. The data indicated a significant decrease in the number of living cells and about 7%–9% reduction in A375.S2 cells treated with 150–200 nM casticin when compared to control groups. However, 100–125 nM of casticin did not significantly reduce the total viable cells of A375.S2. Based on these findings, we have used casticin at concentrations of 100–200 nM in all subsequent experiments.

### 2.2. Casticin Inhibits the Motility of A375.S2 Cells

**Figure 2:**
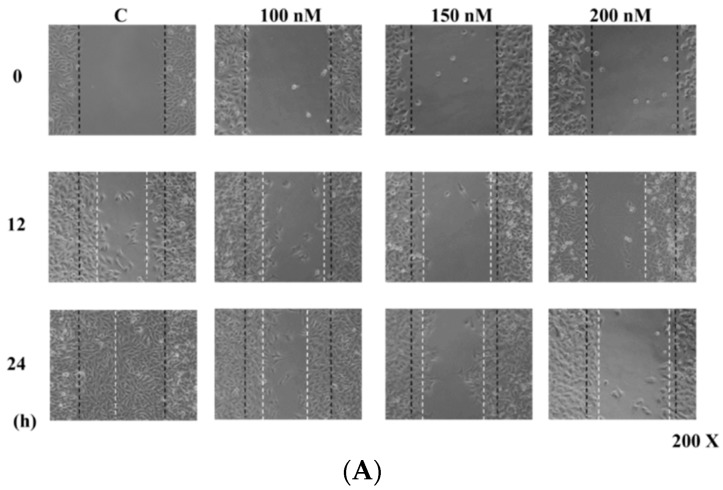

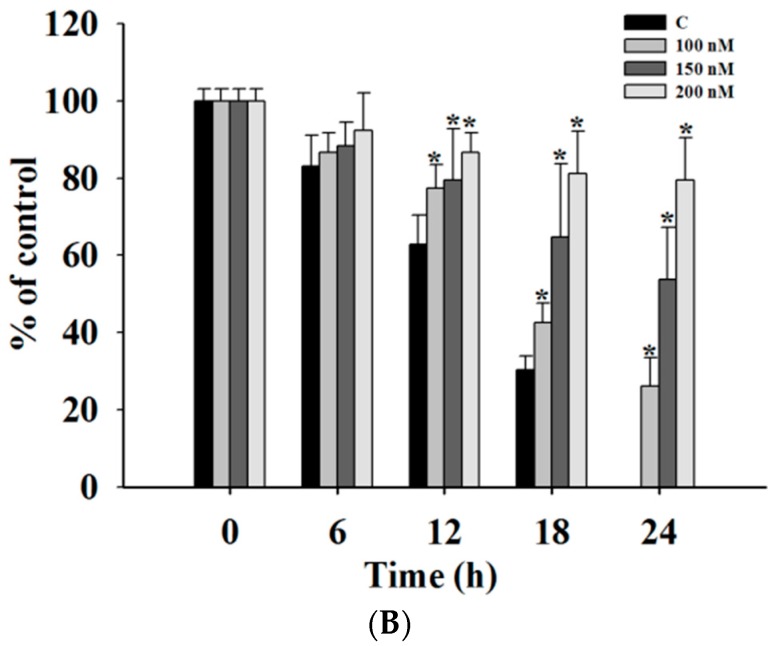
Casticin inhibits the mobility of A375.S2 cells**.** Cells (2.5 × 10^5^ cells/well) were placed into a 6-well plate for confluent monolayer formation in complete medium. Cells in monolayers were wounded by a sterile P200 micropipette tip and remaining cell monolayers were incubated in the medium containing 0, 100, 150 and 200 nM of casticin for 24 h. At the indicated time (0, 6, 12, 18 and 24 h) after scraping, the wound areas were photographed (**A**) and the percentage of cell migration inhibition (**B**) were calculated as described in the Materials and Methods Section. * *p* < 0.05, significant difference between casticin-treated groups and the control as analyzed by Student’s *t* test.

In order to investigate whether casticin inhibits A375.S2 cell mobility, a wound healing (cell migration) assay was performed and results are shown in Figure 2, where continuous rapid movement of A375.S2 cells in a scratch wound assay was found in the control group. However, with 100, 150 and 200 nM casticin treatment, the migration of A375.S2 cells was significantly reduced in a concentration-dependent manner (Figure 2B).

### 2.3. Casticin Inhibit Adhesion of A375.S2 Cells

**Figure 3:**
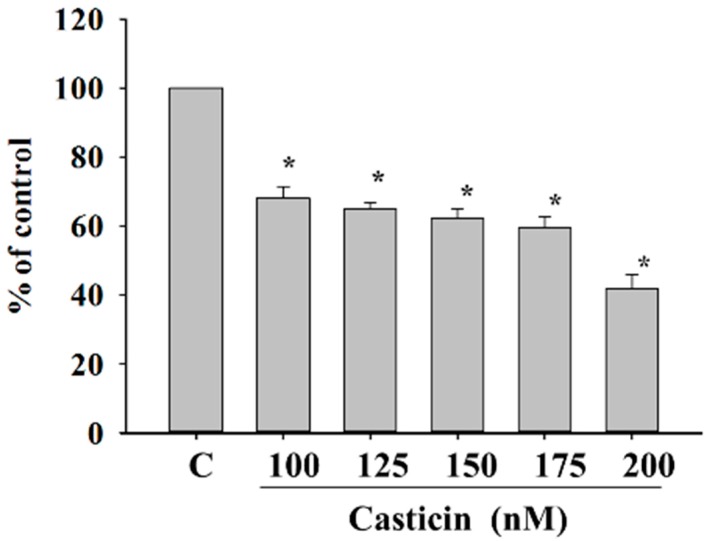
Casticin inhibits the adhesion of A375.S2 cells. Cells (5 × 10^4^ cells/well) plated in 12-well plate were incubated with casticin (0, 100, 125, 150, 175 and 200 nM) for 24 h, unattached cells were removed, and attached cells were mixed in 4% paraformaldehyde and were stained with 0.02% crystal violet solution for 10 min at room temperature. Then DMSO was used to dissolve crystal violet, and O.D. was measured at 570 nm by using microplate reader as described in the Materials and Methods section. Percentage of adhesion was calculated based on the adhesion cells compared to the control. * *p* < 0.05, significant difference between casticin-treated groups and the control as analyzed by Student’s *t* test.

Cancer cell adhesion had been recognized to be a crucial step during cancer invasiveness. Thus, we investigated the effect of casticin on cell adhesion and the results are shown in Figure 3. The data demonstrated that pre-treatment of A375.S2 cells with casticin for 24 h significantly inhibited cell adhesion. Fewer casticin-treated cells adhered to fibronectin than casticin-untreated cells and these effects are dose-dependent, which indicates that the adhesion ability of A375.S2 cells was inhibited by casticin treatment.

### 2.4. Casticin Inhibited the Cell Migration and Invasion of A375.S2 Cells

**Figure 4:**
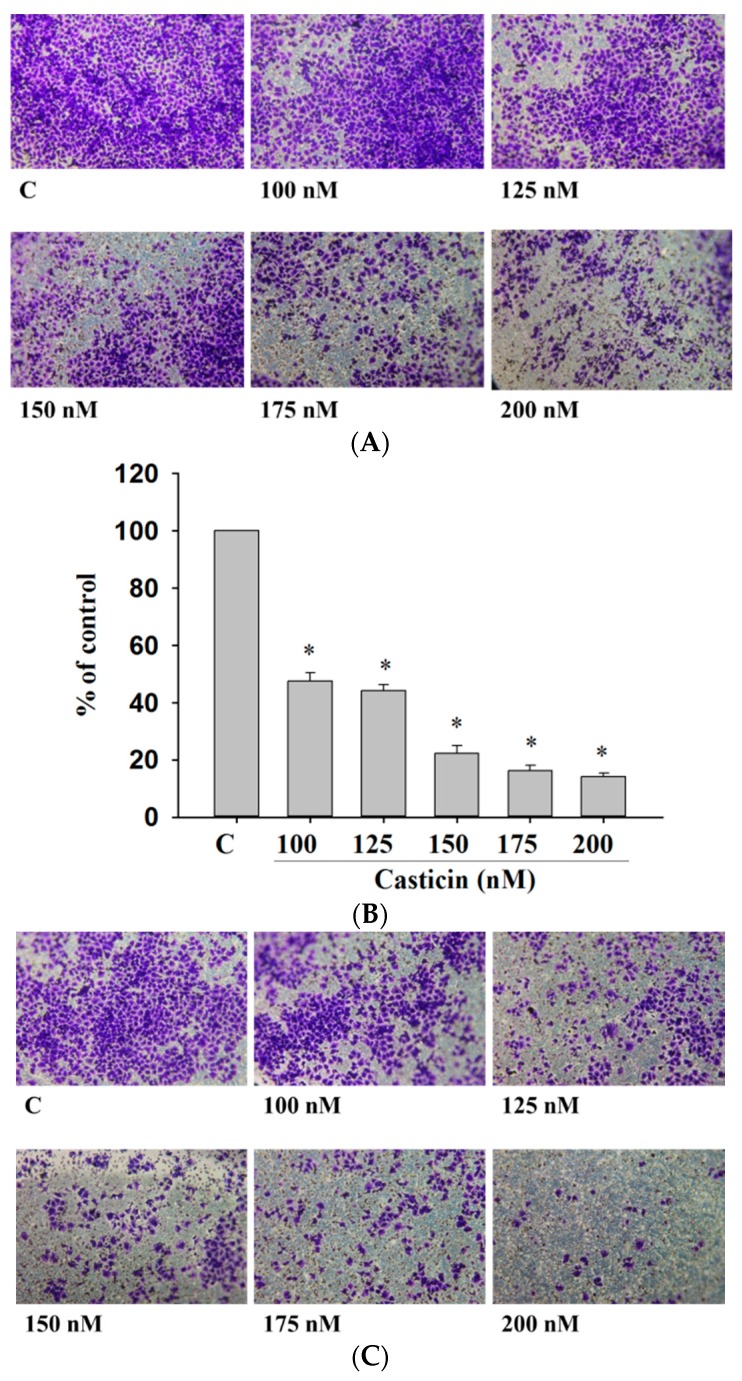

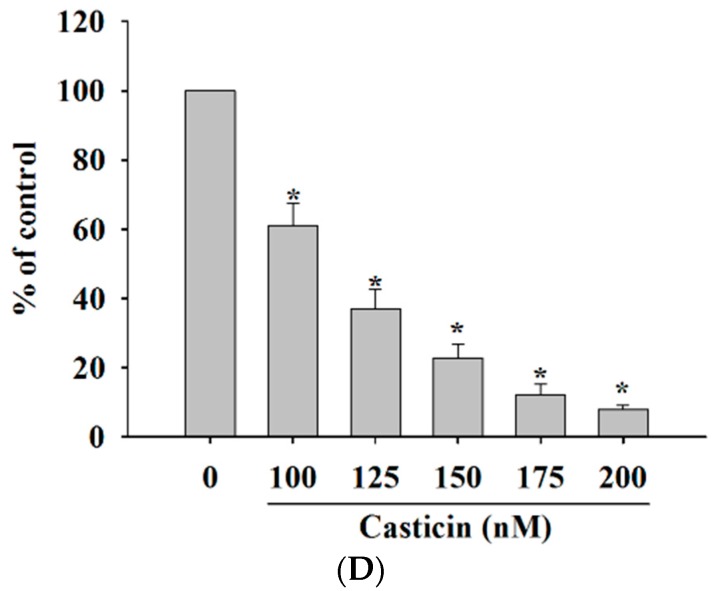
Casticin suppressed the migration and invasion of A375.S2 cells *in vitro*. Cells (2 × 10^4^ cells/well) were placed on filters which were coated with collagen and incubated with casticin (0, 100, 125, 150, 175 and 200 nM) for 24 h. Cells penetrated through to the lower surface of the filter which were stained with crystal violet and were photographed under a light microscope at ×100 (**A**) and the cells were counted (**B**); Cells (2 × 10^4^ cells/well) that penetrated through with the matrigel to the lower surface of the filter were stained with crystal violet and were photographed under a light microscope at ×100 (**C**) and cells were counted (**D**). Results were obtained from three independent experiments. * *p* < 0.05, significant difference between casticin-treated groups and the control as analyzed by Student’s *t* test.

Cell migration and invasion are involved and play important steps in cancer metastasis. Therefore, the inhibitory effects of casticin on A375.S2 cell migration and invasion were measured by a Transwell cell migration and invasion assays and the results are shown in Figure 4. Treatment of A375.S2 cells with increasing concentrations of casticin led to a dosage-dependent decrease in cell vertical migration through the Transwell chamber (Figure 4A,B). Casticin significantly inhibited *in vitro* cell migration by 40% and 93% for 100 and 200 nM at 24 h compared to control cells (Figure 4A,B). We obtained similar results in a wound healing assay where casticin inhibited the *in vitro* mobility of A375.S2 cells (Figure 3). Cell invasion was measured by Transwell matrigel invasion assay and results shown in Figure 4C,D. That indicated that the invasive ability of A375.S2 cells was reduced by casticin treatment and these effects are ocurr a dose-dependent manner (Figure 4C,D). Casticin significantly inhibited *in vitro* cell invasion by 56% and 88% for 100 and 200 nM at 24 h compared to control cells (Figure 4D).

### 2.5. Casticin Downregulates the Proteolytic Activity and Protein Expression of MMP-2

**Figure 5:**
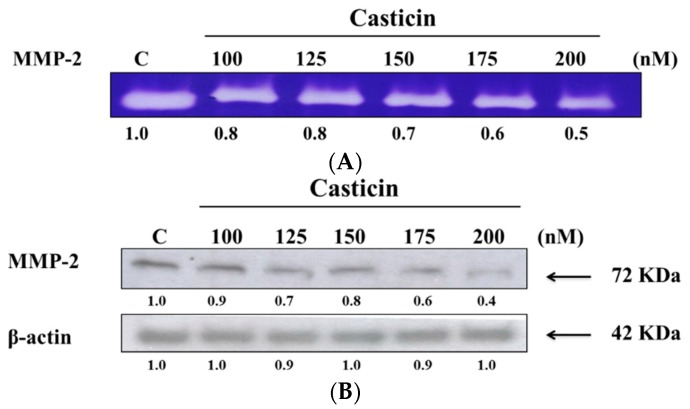
Casticin inhibited the enzyme activities and protein expression of MMP-2 in A375.S2 cells. Cells (2.5 × 10^5^ cells/well) were plated in 6-well tissue culture plates and treated with different concentrations of casticin (0, 100, 125, 150, 175 and 200 nM). The culture medium from treated cells was collected and 50 μg of medium were separated on 8% sodium dodecyl sulfate (SDS)-polyacrylamide gel polymerized with 0.19% gelatin as described in the Materials and Methods section. MMP-2 activity was visualized as clear bands against the blue-stained gelatin background (**A**); MMP-2 protein was quantitated and assayed by Western blotting (**B**) as described in the Materials and Methods section.

Gelatin zymography and western blotting assay were employed to assess the effects of casticin on the activity of secreted MMP-2 and protein expression, respectively, and the results are shown in Figure 5. Figure 5A indicates the appearance and intensity of clear bands which reflect the fact that the proteolytic activity of MMP-2 was reduced upon exposure to casticin in a dose-dependent manner. At 200 nM of casticin, the activity of MMP-2 was reduced by 50% as compared to the untreated control (Figure 5A). Furthermore, the western blotting results indicated that casticin inhibited the protein expression of MMP-2 in a dose-dependent manner (Figure 5B) and at 200 nM of casticin the protein expression of MMP-2 was reduced by 60% as compared to the untreated control (Figure 5B).

### 2.6. Casticin Inhibits the Binding and Protein Expression of NF-κB

**Figure 6:**
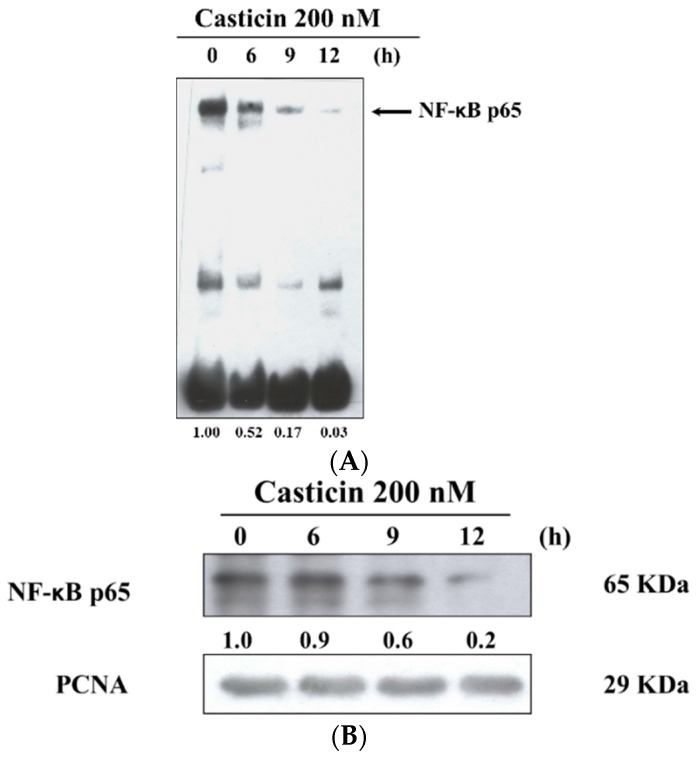
Casticin inhibited the NF-κB p65 binding on the promoted of NF-κB p65 and also inhibited NF-κB p65 protein expression in A375.S2 cells. 1 × 10^6^ cells in 10 cm dish were treated with 200 nM casticin for 0, 6, 9 and 12 h. Nuclear extracts were prepared using the NE-PER Nuclear and Cytoplasmic Extraction kit and quantitated protein concentrations and 5′-biotin-GATCCAGGGGACTTTCCCTAGC-3′ corresponding to the consensus site of NF-κB p65 was used as biotin end-labeled oligonucleotide sequences. EMSA was used for measuring NF-κB p65 binding on the promoter of NF-κB p65 (**A**) as described in the Materials and Methods section, or protein was used to do western blotting for measuring protein expression of nuclear NF-κB p65 (**B**).

After A375.S2 cells were treated with 200 nM casticin for 0, 6, 9 and 12 h, cells were then harvested for examining the binding of NF-κB p65 promoter and protein expression of NF-κB p65 which were assayed by EMSA and western blotting, respectively, and the results are shown in Figure 6. The results indicated that casticin inhibited the binding of NF-κB p65 promoter by NF-κB p65 and these effects are time-dependent (Figure 6A). Furthermore, the protein expression of NF-κB p65 in nuclear fraction was inhibited by casticin in a time-dependent manner (Figure 6B).

### 2.7. Casticin Alters the Levels of Proteins Associated with Migration and Invasion of A375.S2 Cells

**Figure 7:**
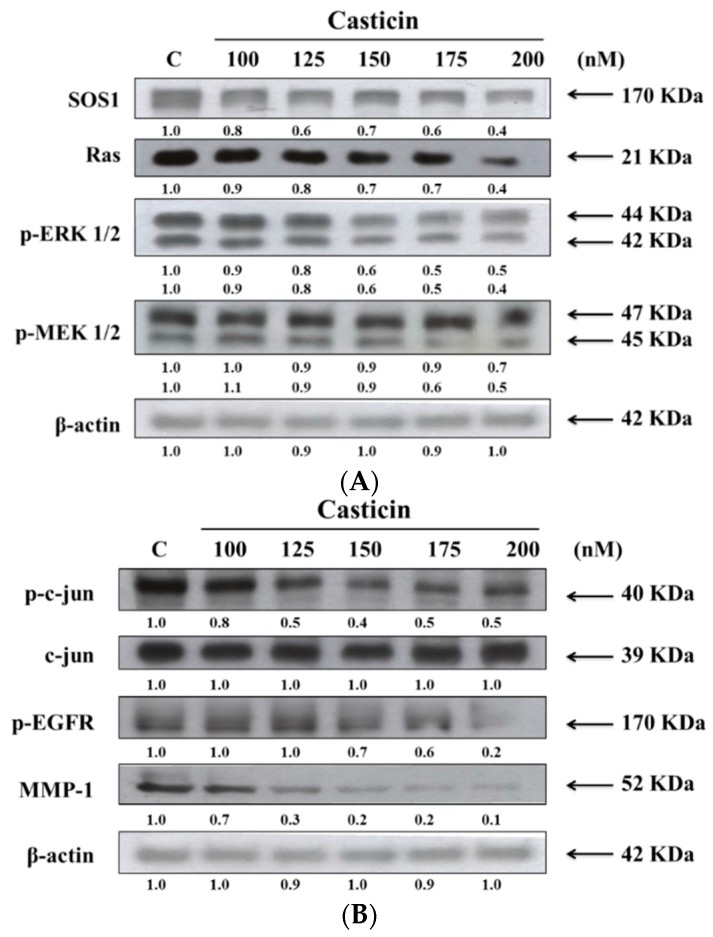
Casticin affect the levels of associated proteins in migration and invasion of A375.S2 cells. Samples were obtained from nuclear extract preparation or A375.S2 cells (1 × 10^6^ cells/dish) were kept in 10 cm dish and were treated with 0, 100, 125, 150, 175 and 200 nM of casticin for 24 h. Each total cell lysate was prepared for western blotting assay as described in the Materials and Methods Section and after blocking with 5% nonfat skim milk, the membrane was probed with primary antibodies for SOS1, Ras, p-ERK 1/2and p-MEK 1/2 (**A**), p-c-jun, c-jun, p-EGFR and MMP-1 (**B**).

We have shown that casticin inhibited the cell migration and invasion of A375.S2 cells, thus, we further investigated whether casticin inhibited cell migration and invasion through the suppression of proteins which are associated with cell migration and invasion. The results from western blotting are shown in Figure 7.

As indicated in Figure 7, casticin treatment resulted in a reduction of SOS1, Ras, p-ERK 1/2 and p-MEK 1/2 (Figure 7A), p-c-jun, p-EGFR and MMP-1 (Figure 7B) but did not affect c-jun in A375.S2 cells. These results showed that the inhibition of those associated with migration and invasion proteins expressions may be involved in reduced expression of MMP-2 and reduced A375.S2 cell migration and invasion.

## 3. Discussion

It is well known that cancer metastasis is a complex process involving cell migration, adhesion and invasiveness [32]. Cancer invasion depends on the action of extracellularly secreted proteases such as MMPs to degrade the basement membrane barrier and to assist cancer cells to escape and migrate. The inhibition of MMPs secretion in cancer cells may be an effective strategy for preventing cancer cell migration and invasion. Melanomas are types of skin tumors that generally have a poor cure rate because of their invasive behavior. The treatments for melanoma include surgery, radiotherapy, and chemotherapy. In the present study, we investigated the role of casticin (a naturally occurring plant phytochemical) in suppressing cell growth, migration, adhesion and invasion of human melanoma A375.S2 cells *in vitro*. Several previous studies have reported that casticin displayed cytotoxic effects on many human cancer cell lines through different molecular pathways, including caspase-dependent and mitochondria-dependent pathways [22,23,28], whereas there is no available information on the effects of casticin on the inhibition of cancer cell migration and invasion of human melanoma cells.

Our results showed that casticin exhibited dose-dependent toxicity towards A375.S2 cells. This observation is in agreement with the cytotoxic property of casticin reported in other findings [21,22,23,24]. Nonetheless, little is known about the anti-metastatic potential of casticin. Hence, the migration and invasion potential of A375.S2 cells were measured with treatments of casticin at subtoxic concentrations (100–200 nM). We demonstrated for the first time that casticin, at subtoxic concentrations, could inhibit the metastasis of A375.S2 cells in a dose-dependent manner (Figure 2). Casticin inhibited cell migration by 40% and 93% for 100 and 200 nM at 24 h compared to control cells (Figure 4). Casticin inhibited cell invasion by 56% and 88% for 100 and 200 nM at 24 h compared to control cells (Figure 4C,D).

Thus far, the detailed mechanism by which casticin exerts inhibitory effects against the migration and invasion of A375.S2 cells is still unclear. High MMP expression has been shown to correlate with the invasive and aggressive behavior of tumors. These MMPs are secreted from cancer cells to degrade the ECM proteins in the basement membrane barrier which eventually promotes the migration and invasion behavior of the cancer cells [11,33]. MMP-2 and -9 are the gelatin enzymes which have been reported to degrade the matrix collagen and basement membrane [34,35]. It was reported that multiple signaling pathways are associated with the regulation and expression of MMPs, including the MAPK and NF-κB signaling pathways [36,37]. If agents can block one of the pathways, it may lead to inhibition of cancer metastasis.

In the present study, we demonstrated that MMP-2 activity in conditioned serum-free medium of A375.S2 cells was significantly downregulated by casticin (Figure 5). At 200 nM of casticin, the activity of MMP-2 was reduced by 50% as compared to the untreated controls (Figure 5). This observation may indicate that the inhibition of cell invasion and migration by casticin could be mediated by the reduction in the proteolytic action of MMP-2. The results from western blotting experiments also showed that casticin reduced the protein expression of MMP-2 (Figure 5B) and MMP-1 (Figure 7B) in A375.S2 cells. In the degradation of the basement membrane, MMPs, especially the gelatinolytic MMP-2, is one of enzymes which plays a central role [38]. It was also reported that oxidative stress can drive RPE cells to strongly express MMP-1. MAPKs activation also involved the critical increased of expression of MMP-1 in EGF-induced glioma invasion [39]. Results from western blotting (Figure 7A,B) indicated that casticin suppressed the expression of p-ERK 1/2, p-MEK 1/2, p-c-jun and p-EGFR in A375.S2 cells. Thus, we suggest casticin inhibited cell migration and invasion of A375.S2 cells through the inhibition of MMP-2 and -1.

Active NF-κB signaling pathways are usually found in highly invasive cancer cells [40] and blocking NF-κB signaling pathways can result in decreased breast cancer cell migration [41]. NF-κB had been shown to play a role in the immuno-inflammatory response, especially in numerous skin diseases and skin cancer and it consists of dimeric p65 and p50 transcription factors [42]. NF-κB plays a role in initiation, development and promotion of skin carcinomas [42]. Herein, we found that casticin significantly inhibited the binding of NF-κB p65 on NF-κB p65 promoter which was assayed by EMSA (Figure 6A) and it also decreased the protein levels of NF-κB p65 which was measured by western blotting (Figure 6B). Furthermore, the results from Figure 7 also show that casticin decreased the expression of p-ERK 1/2, p-MEK 1/2, p-c-jun, p-EGFR and MMP-1 in A375.S2 cells, indicating that casticin inhibited cell migration and invasion and this involved NF-κB p65, p-EGFR and p-ERK 1/2 in A375.S2 cells *in vitro*.

## 4. Materials and Methods

### 4.1. Chemicals and Reagents

The natural compound casticin, dimethyl sulfoxide (DMSO) and propidium iodide (PI) were purchased from Sigma-Aldrich Corp. (St. Louis, MO, USA). Casticin formed yellow crystals, and has a molecular weight of 374.34 with a purity of 99.0%. Casticin was dissolved in 0.1% DMSO and then diluted with phosphate-buffered saline (PBS). Cell culture materials such as MEM medium, fetal bovine serum (FBS), l-glutamine and penicillin-streptomycin were purchased from GIBCO^®^/Invitrogen Life Technologies (Carlsbad, CA, USA). Primary antibodies against MMP-1 and -2, NF-κB p65, SOS1, Ras, p-ERK 1/2, p-MEK 1/2, p-c-jun, c-jun and p-EGFR and secondary antibodies were obtained from Cell Signaling Technology, Inc. (Beverly, MA, USA). The enhanced chemiluminescence (ECL) detection system was obtained from Amersham Life Sciences, Inc. (Arlington Heights, IL, USA).

### 4.2. Cell Culture

Human melanoma A375.S2 cell line was obtained from the Food Industry Research and Development Institute (Hsinchu, Taiwan). Cells were cultured in MEM supplemented with 10% fetal bovine serum (FBS), 2 mM l-glutamine and antibiotics (100 units/mL penicillin, 100 μg/mL streptomycin) at 37 °C incubator with a humidified atmosphere of 95% air and 5% CO_2_ [43]. 

### 4.3. Cell’s Viability Assays

A375.S2 cells (5 × 10^4^ cells) were plated in a 12-well plate for 24 h and after the required confluence was reached, cells were incubated with various concentrations of casticin (0, 100, 125, 150, 175 and 200 nM) or 0.5% DMSO as a vehicle for 24 h. Cells were harvested from each treatment, counted and stained with PI (5 μg/mL) and then were immediately analyzed by using flow cytometer (BD Biosciences, Bedford, MA, USA) assay for the percentages of viability as previously described [10].

### 4.4. Wound Healing Mobility Assay

To determine the effect of casticin on migration of A375.S2 cells *in vitro*, cells were seeded at 2.5 × 10^5^ cells/well in 6-well plate and grown to about 90% confluence after 24 h. Medium was removed and cell monolayers were wounded by manually scraping the cells by a sterile P200 micropipette tip. Debris was washed off with PBS three times and cells were then cultured in serum-free medium containing 0, 100, 150 and 200 nM of casticin for 24 h at 37 °C and the wound areas were photographed [10].

### 4.5. Adhesion Assay

A375.S2 cells (5 × 10^4^ cells/well) were plated in 12-well plate for 12 h and then were incubated with casticin (0, 100, 125, 150, 175 and 200 nM) for 24 h. Cells were harvested from each treatment, and reseed in the 24-well for 3 h. Rinse cells three times with PBS and then fixed cells with 4% paraformaldehyde at room temperature for 15 min. Add 0.02% crystal violet solution to stain cells for 10 min at room temperature, and then added DMSO to dissolve crystal violet. The O.D. was measured at 570 nm by using microplate reader (Bio-Rad Laboratories, Hercules, CA, USA). Percentage of adhesion was calculated based on the adhesion cells compared to the control [44].

### 4.6. Cell Migration and Invasion Assay

For cell migration and invasion assays, A375.S2 cells (2 × 10^4^ cells/well) were placed in Transwell cell culture chambers (8 mm pore size; Merck Millipore Corp., Billerica, MA, USA), which were coated with collagen for migration assay or matrigel for invasion assay for 24 h. Cell suspension was placed in the upper chamber of the Transwell insert and incubated with 0.5% DMSO as control or casticin (0, 100, 125, 150, 175 and 200 nM), and lower chamber was filled with complete medium (90% MEM medium containing 10% FBS) as a chemoattractant. Cells were incubated for 24 h and this led cells to migrate or invasive. Migrated or invasive cells in membrane were fixed with 4% formaldehyde and then were stained with 0.02% crystal violet as described previously [44]. Cells were examined, photographed and quantified under a light microscope at 100× in five random fields per membrane. Each sample was assayed in triplicate as described previously [44].

### 4.7. Gelatin Zymography Assay

The enzyme activities of MMP-2 were analyzed by gelatin zymography assay. A375.S2 cells (2.5 × 10^5^ cells/well) were plated in 6-well plates and then culture medium was replaced by serum-free MEM medium (1 mL per well) and treated with different concentrations of casticin (0, 100, 125, 150, 175 and 200 nM). A 50 μL of medium were separated on 8% sodium dodecyl sulfate (SDS)-polyacrylamide gel polymerized with 0.19% gelatin. After electrophoresis, the gels were washed with ddH_2_O and incubated in a renaturing buffer (2.5% Triton X-100 in ddH_2_O, 3 times, 15 min). After renaturing, incubated gel in the developing buffer (50 mM·Tris-HCl, 150 mM·NaCl, 10 mM·CaCl_2_, 1 μM·ZnCl_2_, 0.02% NaN_3_, pH 7.5) for 24 h at 37 °C. After incubation, the gel was stained with 1.5% Coomassie R-250 and then destained with 10% acetic acid in 30% methanol. MMP-2 activity was visualized as clear bands against the blue-stained gelatin background [45].

### 4.8. Nuclear Extract Preparation.

The A375.S2 cells (1 × 10^6^ cells/dish) in 10 cm dish were treated with 200 nM casticin for 0, 6, 9, and 12 h and then cells were harvested. Cells from each time periods were fractionated into cytoplasmic and nuclear extracts using Nuclear Extraction Kit.

### 4.9. Western Blotting Assay

Samples were obtained from nuclear extract preparation or A375.S2 cells (1 × 10^6^ cells/dish) were kept in 10 cm dish and were treated with 0, 100, 125, 150, 175 and 200 nM of casticin for 24 h. Each total cell lysate was prepared in a lysis buffer (40 mM·Tris-HCl (pH 7.4), 10 mM EDTA, 120 mM NaCl, 1mM dithiothreitol, 0.1% Nonide P-40) and quantitated by Bradford protein assay. Using BSA as standard. Equal amounts of protein (30 μg) were separated on 10% sodium dodecyl sulphate (SDS)-polyacrylamide gel for electrophoresis and transferred onto a polyvinylidene fluoride (PVDF) membrane (Millipore, Temecula, CA, USA) by electro-blotting at 400 mA for 90 min. After blocking with 5% nonfat skim milk, the membrane was probed with primary antibodies for NF-κB p65, MMP-2, SOS1, Ras, p-ERK 1/2, p-MEK 1/2, p-c-jun, c-jun, p-EGFR, MMP-1, β-actin and specific secondary antibodies. The protein expression was detected by ECL solution (Millipore) as described previously [43,44,45].

### 4.10. Electrophoretic Mobility shift Assay (EMSA)

1 × 10^7^ cells in 10 cm dish were treated with casticin (0 and 200 nM) for 6, 9 and 12 h. Nuclear extracts were prepared using the NE-PER Nuclear and Cytoplasmic Extraction kit (Pierce, Rockford, IL, USA) as described previously [44]. Quantitated protein concentrations and 5′-Biotin-GATCCAGGGGACTTTCCCTAGC-3′ corresponding to the consensus site of NF-κB was used as biotin end-labeled oligonucleotide sequences. A 5 μg of nuclear extract proteins were used for EMSA with LightShift Chemiluminescent EMSA Kit based on the manufacturer’s protocol. Biotin end-labeled duplex DNA was incubated with nuclear extracts or purified factor and were electrophoresed on a 6% polyacrylamide native gel. After electrophores, DNAs were transferred to a positive nylon membrane, UV cross-linked, probed with streptavidin-HRP conjugate and incubated with the substrates of the ECL kit as described previously [44].

### 4.11. Statistical Analysis

All data are presented as mean ± S.D from at least three experiments. All statistics were calculated by Student’s *t*- test using Sigma plot software (software version 10.0, Systat Software Inc., San Jose, CA, USA) and a *p* value of less than 0.05 was considered statistically significant.

## 5. Conclusions

In summary, casticin decreased cell migration, adhension, invasion and expression of ECM-associated proteases in human melanoma A375.S2 cancer cells *in vitro*. Casticin suppressed the expressions of the MAPK and NF-κB signaling molecules involved in p-ERK 1/2, p-MEK 1/2, p-EGFR, and NF-κB signaling pathways for leading to the inhibition of MMP-2 and -1 in A375.S2 cancer cells. These findings strongly support the development of casticin as an anti-metastatic agent and an alternative therapeutic approach to combat human melanoma cell metastasis in the future.
